# Editorial: Evolution of Animal Microbial Communities in Response to Environmental Stress

**DOI:** 10.3389/fmicb.2022.860609

**Published:** 2022-03-30

**Authors:** Teresa Nogueira, Ana Botelho, Lucas Bowler, João Inácio

**Affiliations:** ^1^Bacteriology and Mycology Laboratory, Instituto Nacional Investigação Agrária e Veterinária (INIAV), Oeiras, Portugal; ^2^Centro de Ecologia, Evolução e Alterações Ambientais (cE3c), Universidade de Lisboa, Lisbon, Portugal; ^3^School of Applied Sciences, University of Brighton, Brighton, United Kingdom

**Keywords:** microbiomes, adaptation, ecology and population biology, environment, stress response, microbial evolution, microbiome and dysbiosis, microbiome in health and disease

We live in a microbial world in which bacteria, viruses, fungi, and other microscopic organisms such as parasites live together in microbial communities, where they establish ecological interactions, cooperative and competitive behaviors, driving to environmental adaptation and genome evolution.

A microbiome represents the collection of the microorganisms that inhabit a specific environment along with their genomic content. They are present everywhere and in many niches of the human body. Metagenomics brings together microbiology, genetics, genomics, and bioinformatics, enabling an integrated approach to the understanding of the natural microbial communities to capture the ecology, evolution, and dynamics of these microbial communities.

In a changing environment, microorganisms evolve and adapt, and the balance between species and strains within the community can also be disrupted or shifted to another level of steady-state. Human and other animal microbiomes can be under diverse sources of disturbance: physiological changes due to diet or health/illness, exposure to medications, or even environmental changes, among others. To understand and study the effect of these changes on animal microbial communities' dynamics, the most appropriate sampling design tools must be applied, and mathematical and computational methods must also be developed to deal with this challenging subject. This Research Topic “*Evolution of Animal Microbial Communities in Response to Environmental Stress*” addresses the dynamics of microbial (symbiotic) communities under environmental changes.

The microbiome of individuals who share the same household or social contacts is more likely to share similar microbes (Brito et al., 2019). Thus, cohabitation has been reported to be a driver of the transfer and sharing of the oral, gut, and nasopharyngeal microbiomes from person to person in a physical social network, as well as, for example, a major factor facilitating the typical asymptomatic transmission of SARS-CoV-2 (Hu et al., 2020). Chen Y. et al. conducted a confined experiment in which healthy individuals were enclosed together in a space capsule on the ground for 180 days, which revealed that both the structure and diversity of the nasal and oropharyngeal microbiota changed over time and were shared between the participants, during the study. A work by Fu et al. shows that domestication is also a modulator of gut microbiome diversity in yak which paves the way for the development of new commercial strains in livestock based on the biotechnology of gut microbiota.

The microbial community of the Qinghai-Tibet Plateau (QTP) includes parasites of mammalian hosts, such as some rodent species. Wu et al. have demonstrated that the altitude of the Qinghai-Tibet Plateau is a driver of diversity, not only of plants and animals but also of microorganisms such as parasites and their microscopic larvae of Taeniidae species.

A classic example of the adaptive response of populations and communities is exposure to antibiotics. The study by Chen C.-M. et al. demonstrates that changes in the gut microbiota following maternal exposure to antibiotics during pregnancy can be transmitted to offspring, also leading to a disruption in gut development in neonatal mice.

Dietary modification can also have an effect as a modulator of the gut microbiota, inducing the appearance of new genomic variants in the gut microbiome. For example, one study shows that changing the diet to one rich in fiber, in children with Prader-Willi syndrome and obese children, led not only to changes in microbial diversity but also to the evolution of the microbial genome with the appearance of variants corresponding to SNPs related to traits of adaptation to the use of nutrients from the diet, thus opening the prospect of investigating new therapeutic pathways (Li et al.). Diet is also involved in modeling gut microbiota and the immune stimulation of sows, which may reduce the diarrhea rate of their offspring (Zhang et al.).

The human gut is an ecosystem comprising trillions of microbes that reacts to diet and interacts with the host influencing its health. Close relationships between dietary modifications, microbiota composition and health status have been established (Enam and Mansell, 2019). Furthermore, gut microbes develop catabolic activity to circumvent the antimicrobial activity of certain food additives such as vanillin. Gene clusters specific for the vanillin metabolism and intermediary metabolic pathways have been identified by metagenomic analysis in several gut microbial populations (Yadav et al.). The metabolomic analysis brings forth the functionality of the vanillin catabolic pathway. These results highlight the human gut microbial features and metabolic bioprocess involved in vanillin catabolism to overcome its antimicrobial activity.

Another example of the relationship between the diversity of the gut microbiome and health is presented by Ma et al. that have demonstrated that there is a correlation between native Tibetans and Han immigrants' gut microbiome and their hematological profile, which may also relate to the Tibetans' adaptation to high altitudes.

The determination of the vaginal microbiome in women undergoing different types of treatments and the correlation with clinical outcomes have been the object of several studies (Hyman et al., 2012). The changes in the composition of the vaginal microbiome under the effect of menopausal hormonal therapies consist in a decrease in microbial diversity and a preponderance of *Lactobacillus* (Geng et al.). In contrast, the non-treated vaginal microbiome is enriched with anaerobic and facultative anaerobic bacteria, namely, *Gardnerella, Prevotella, Escherichia, Shigella, Streptococcus, Atopobium, Aerococcus, Anaerotruncus, and Anaerococcus*, apart from *Chlamydia* and *Streptococcus*. These changes have consequences in the symptoms of the genitourinary syndrome in menopause that are more severe without the effect of hormonal therapies.

It is known that the natural respiratory tract microbiome plays an important role in controlling nosocomial infections. However physiological conditions and interventions of critical respiratory tract patients, such as mechanical ventilation and antibiotics administration, dramatically alter the respiratory tract microbiome, leading to dysbiosis and favoring the colonization by opportunistic and resistant pathogens (Pérez-Cobas et al.). Therefore, this microbial imbalance is linked with various diseases and significantly reduces the quality of one's life. Varying inflammatory profiles are associated with specific microbial compositions, while the same is true for many disease conditions and environmental exposures. A shift in the microbial composition is also detected upon the administration of numerous therapeutics, highlighting other beneficial and adverse side effects (Elgamal et al., 2021).

The commensal bacteria that show the potential to inhibit pathobionts and modulate host immunity should be further studied for their potential to stimulate a resilient microbiota that is resistant to infection. The development of *in vivo* and *in vitro* models that assess microbial competition and interactions within the microbiota will further the understanding of the complex relationships that exist and will help develop probiotic solutions for respiratory tract infections.

## Author Contributions

TN, AB, LB, and JI contributed to conception of the aim of the Research Topic. TN and AB wrote the manuscript. All authors contributed to manuscript revision, read, and approved the submitted version.

## Funding

Fundação para a Ciência e a Tecnologia (FCT), I.P., supports TN by contract ALG-01-0145-FEDER-028824 and PTDC/BIA-MIC/28824/2017 and cE3c-FCUL through contract UIDP/00329/2020.

## Conflict of Interest

The authors declare that the research was conducted in the absence of any commercial or financial relationships that could be construed as a potential conflict of interest.

## Publisher's Note

All claims expressed in this article are solely those of the authors and do not necessarily represent those of their affiliated organizations, or those of the publisher, the editors and the reviewers. Any product that may be evaluated in this article, or claim that may be made by its manufacturer, is not guaranteed or endorsed by the publisher.
